# Silencing of Double-Stranded Ribonuclease Improves Oral RNAi Efficacy in Southern Green Stinkbug *Nezara viridula*

**DOI:** 10.3390/insects12020115

**Published:** 2021-01-28

**Authors:** Rohit Sharma, Clauvis Nji Tizi Taning, Guy Smagghe, Olivier Christiaens

**Affiliations:** Department of Plants and Crops, Faculty of Bioscience Engineering, Ghent University, Coupure Links 653, B-9000 Ghent, Belgium; rohit.sharma@ugent.be (R.S.); tiziclauvis.taningnji@ugent.be (C.N.T.T.); guy.smagghe@ugent.be (G.S.)

**Keywords:** dsRNA, *N*. *viridula*, *NvdsRNase*, oral feeding bioassay, “RNAi-of-RNAi”, saliva and midgut juice

## Abstract

**Simple Summary:**

RNAi interference (RNAi) is a conserved mechanism found in all eukaryotes. This mechanism is initiated by the presence of double-stranded RNA in the cells and leads to the blockage of protein synthesis of a target gene. This technique is being explored to develop species-selective biopesticides, where insect-specific double-stranded RNA would be delivered to an insect via the oral route. However, orally delivered double-stranded RNA leads to a variable RNAi-interference efficacy in different insect orders. Previous studies have shown rapid degradation of double-stranded RNA in the saliva of the southern green stinkbug. In this study, we identified and characterized the protein associated with double-stranded RNA degradation and provided evidence of the involvement of this protein in limiting RNAi efficacy in this pest. Our results revealed that one protein, a double-stranded RNA nuclease, is associated with double-stranded RNA degradation. Further, the blockage of double-stranded RNA nuclease synthesis by RNAi-interference significantly enhances the death-rate in the southern green stinkbug. These findings will be useful in the development of RNAi-interference-based pest control strategies.

**Abstract:**

Variability in RNA-interference (RNAi) efficacy among different insect orders poses a big hurdle in the development of RNAi-based pest control strategies. The activity of double-stranded ribonucleases (dsRNases) in the digestive canal of insects can be one of the critical factors affecting oral RNAi efficacy. Here, the involvement of these dsRNases in the southern green stinkbug *Nezara viridula* was investigated. First, the full sequence of the only dsRNase (*NvdsRNase*) in the transcriptome of *N*. *viridula* was obtained, followed by an oral feeding bioassay to evaluate the effect of *NvdsRNase*-silencing on oral RNAi efficacy. The *NvdsRNase* was first silenced in nymphs by *NvdsRNase*-dsRNA injections, followed by exposure to an artificial diet containing a lethal *αCop*-specific dsRNA. A significantly higher mortality was observed in the *NvdsRNase-*silenced nymphs when placed on the ds*αCop*-containing diet (65%) than in the ds*GFP* injected and ds*αCop* fed control (46.67%). Additionally, an ex vivo dsRNA degradation assay showed a higher stability of dsRNA in the saliva and midgut juice of *NvdsRNase-*silenced adults. These results provide evidence for the involvement of *NvdsRNase* in the reduction of oral RNAi efficacy in *N*. *viridula*. This information will be useful in further improving potential RNAi-based strategies to control this pest.

## 1. Introduction

RNA-interference (RNAi) has been exploited in molecular biology for various purposes since the discovery of its mechanism at the end of the 20th century [1]. RNAi has proved to be an excellent tool for functional genomics in various research areas, by allowing loss-of-function analysis of genes in insects, plants and fungi [2,3,4,5,6]. In insects, RNAi-mediated gene silencing has been frequently employed as a reverse genetic tool in the laboratory through injecting target gene-specific double-stranded (dsRNA) into the insects’ haemocoel [7]. DsRNA is subsequently taken up by the target tissue cells and then processed by the core RNAi-machinery, leading to the formation of small interfering RNAs (siRNAs) [7]. After coupling with the RNA-induced silencing complex (RISC), the siRNAs guide the complex to the specific complementary mRNA which is then cleaved in a homology-dependent manner [7]. Due to the species-selectivity and biodegradable active ingredient, this novel mode of action has been actively promoted in the development of biosafe next-generation biopesticides [8,9,10]. For a successful pest control strategy, dsRNA delivery via the oral route is the most practical method. However, RNAi responses to the orally delivered dsRNA are highly variable among insect species [8,11,12,13]. Coleopterans are generally considered to be highly sensitive; on the other hand, other insect orders such as dipterans, hemipterans and lepidopterans show a more variable or low response to the orally delivered dsRNA [2,13,14,15,16,17]. This variation can be attributed to different factors such as dsRNA cellular uptake, dsRNA-nuclease activity, dsRNA length and other factors. [18]. Previous studies have shown that double-stranded ribonuclease (dsRNase) activity in the digestive canal plays a crucial role in limiting the oral RNAi response in various insect orders [13,14,19,20,21]. Although the genes have been found to be expressed in various tissues and fluids, dsRNase activity has notably been shown in the digestive system of insects [12,19,22,23,24], and responsible genes have been identified and characterized in multiple insect species [23,24,25,26,27]. Further, RNAi-mediated silencing of dsRNases, followed by an RNAi-treatment targeting an essential gene (a so-called “RNAi-of-RNAi” assay), led to a significantly enhanced RNAi efficacy for the essential gene in coleopterans (*Tribolium castaneum* [23], *Cylas puncticollis* [25], *Leptinotarsa decemlineata* [28]), orthopterans (*Locusta migratoria* [29]), and the dipteran *Bactrocera tryoni* [26]. Similar results have also been observed in *Bemisia tabaci* (Hemiptera, suborder: Homoptera: Aleyrodidae), and these results suggested that this strategy can be exploited to enhance the RNAi efficacy in sucking pests [30]. However, among sucking pests, in stinkbugs (Hemiptera, suborder: Heteroptera: Pentatomidae), clear evidence of the involvement of these dsRNases in a reduced oral RNAi efficacy has not been provided yet.

Stinkbugs (Pentatomids) are polyphagous pests but mainly infest leguminous crops, especially soybean (*Glycine max*) [31]. Among the stinkbugs, the southern green stinkbug, *Nezara viridula*, is an emerging pest which severely affects soybean yield in the major soybean-producing countries and leads to significant economic losses [32,33,34]. Apart from reducing yield, *N*. *viridula* infestation affects the quality of soybean seeds due to its piercing mouthparts [35]. This species has also been reported as a vector of fungal and bacterial diseases in various leguminous crops [36,37]. In a previous study, we have confirmed that orally delivered dsRNA can trigger the RNAi mechanism in *N*. *viridula*, although not very efficiently [38]. Our results also indicated that dsRNA was rapidly degraded in the insects’ saliva, suggesting that the dsRNase activity is one of the limiting factors that lower the RNAi efficacy in this species [38]. It is still unknown whether one or more dsRNases are involved in dsRNA degradation in *N*. *viridula*. In contrast, in other species of the stinkbug-complex; *Euschistus heros* and *Halyomorpha halys*, dsRNA-degrading nucleases have been identified and are available in the public domain [39]; nonetheless, direct proof of their effect on RNAi efficacy has not been shown in these species.

In this study, the aim was to identify potential dsRNases and further investigate their role in reducing oral RNAi efficacy in *N*. *viridula*. First, a transcriptome-wide search was performed to identify potential candidate dsRNase genes, before evaluating their expression profile in different nymphal stages and different tissues. Next, the identified *dsRNases* would be knocked out by RNAi before evaluating dsRNA stability in saliva and midgut juice and RNAi efficacy in an oral feeding bioassay with an essential gene: *αCop*.

## 2. Materials and Methods 

### 2.1. Insect Rearing

*N*. *viridula* nymphs and adults were taken from the mass rearing colony at the Lab of Agrozoology, Ghent University, Ghent, Belgium. The colony was maintained in an incubator (Panasonic, Oizumi, Gunma, Japan) with controlled laboratory conditions of 60 ± 10% relative humidity and 25 ± 2 °C temperature. The conditions of photoperiod, food supply, and maintenance of the insects were followed as described previously [38].

### 2.2. Identification and Characterization of NvdsRNase

With the aim to identify all potential dsRNases in *N*. *viridula*, DNA/RNA non-specific endonuclease in *E*. *heros* [38], the homologous gene in *H*. *halys* (GeneBank accession: XM_014427061.1), DNA/RNA non-specific nuclease 1, −2 and −4 in *C*. *puncticollis* [25] and dsRNase1, −2, −3 and −4 in *Schistocerca gregaria* [27], were used as query sequences in BLASTn searches against the available sequence-reads in the Sequence Read Archive (SRA) database (SRA, accession number: SRP119668) of *N*. *viridula*. A single set of short-reads were hit with all the queries in BLASTn, and the highest identity was observed with *E*. *heros* and *H*. *halys*. Only one hit was identified in this *N*. *viridula* and to obtain the full length of its ORF, a forward primer was designed based on the homologous sequence of *H*. *hayls*, as the SRA did not cover the full-length mRNA of the DNA/RNA non-specific endonuclease of *E*. *heros* and *H*. *hayls*, and a reverse primer was designed from the sequence read of *N*. *viridula* (primers are shown in Table S1). The putative dsRNase open reading frame (ORF) fragment was then amplified by PCR using *N*. *viridula* cDNA as a template. The PCR was run with *Taq* polymerase (Thermo Fisher, Waltham, MA, USA) and at the following conditions: 2 min at 94 °C; 35 cycles of 30 s at 94 °C, 30 s at 60 °C; 30 s at 72 °C; and 10 min at 72 °C. Subsequently, the PCR product was run on a 1.5% agarose gel for 30 min at 100 V, and the expected band was excised from the gel and purified by using the Wizard^®^ SV Gel and PCR Clean-Up System, Promega (Madison, WI, USA). Further, the PCR product was cloned into a pJET 2.1 vector (CloneJET PCR Cloning Kit, Thermos Fisher Scientific, Vilnius, Lithuania), and subsequently the recombinant pJET 2.1 vector was transformed into *E*. *coli* DH5α competent cells (Invitrogen, Karlsbad, CA, USA). The transformed competent cells were then plated on Luria–Bertani (LB) agar plates and incubated at 37 °C overnight. The positive colonies, identified by the colony PCR, were allowed to grow overnight in the LB growth medium at 37 °C and followed by plasmid extraction using the Wizard^®^ SV Minipreps DNA Purification System (Madison, WI, USA). The full length of the potential dsRNase ORF in the recombinant pJET 2.1 vector was sequenced at LGC Genomics (Berlin, Germany).

ORF identity was confirmed with BLASTx (NCBI) by searching against the non-redundant protein database. In the deduced amino acid sequence, protein domain prediction was made by SMART (http://smart.embl-heidelberg.de/), signal peptides were identified by SignalP-5.0 Server (http://www.cbs.dtu.dk/services/SignalP/), and subcellular localization was identified by GeneScript^®^ WoLF PSORT II (https://www.genscript.com/wolf-psort.html) [40] and Euk-mPLoc 2.0 web tool (http://www.csbio.sjtu.edu.cn/bioinf/euk-multi-2/) [41]. The phylogenetic analysis was performed with MEGA v10.2.2 [42]. Multiple sequence alignments were performed by the MUSCLE algorithm. The maximum likelihood procedure was followed to construct the phylogenetic tree with 1000 bootstrap replicates, and the full-length amino acid sequences of the DNA/RNA non-specific nuclease domain of different species were used. The phylogenetic tree was shaded by iTOL (https://itol.embl.de/).

### 2.3. RNA Isolation, cDNA Synthesis and dsRNA Synthesis

Total RNA was isolated from second-instar nymphs using the RNeasy Mini kit (Qiagen, Hilden, Germany) from homogenized live individuals in an RLT-buffer + β-mercaptoethanol and following the manufacturer’s instructions. Next, cDNA synthesis was performed starting from 500 ng RNA using the Superscript IV kit (Thermo Fisher, Waltham, MA, USA), following the manufacturer’s instructions. Amplification of *NvdsRNase* and *αCop* gene fragments was performed by *Taq* PCR using *N*. *viridula* cDNA as a template at the following conditions: 2 min at 94 °C, 5 cycles of 30 s at 94 °C, 30 s at 60 °C, 30 s at 72 °C, 35 cycles of 30 s at 94 °C, 30 s at 65 °C, 30 s at 72 °C, and 4 min at 72 °C. For amplification of the *GFP* (green fluorescent protein) fragment, a plasmid with *GFP* insert (Genbank ID: NC_011521.1) was used as a template. PCR products were purified by using the Wizard^®^ SV Gel and PCR Clean-Up System, Promega (Madison, WI, USA). DsRNA synthesis was performed using the MEGAscript™ RNAi Kit (Invitrogen, Thermos Fisher Scientific, Vilnius, Lithuania) and elution of the dsRNA from the columns was performed with nuclease-free water. Concentrations of isolated RNA, PCR product and dsRNA were determined with NanoDrop ND-1000 s (Nanodrop Technologies, Wilmington, DE, USA) at 260 nm, and run on gel electrophoresis for 30 min at 100 V to analyze the purity. Geneious Prime^®^ [43] v2020.2.4 software (https://www.geneious.com) was used to design the primers (Supplementary Materials Table S1).

### 2.4. Developmental Stage-Specific and Tissue-Specific Expression of NvdsRNase and RT-qPCR

*NvdsRNase* expression levels were quantified in different developmental stages and tissues of *N*. *viridula* (midgut, salivary glands, head and the remnant body). Each tissue was dissected and isolated from one adult separately, and salivary glands were removed from the gut before total RNA isolation from the midgut.

All the dissection tools were sterilized by 70% ethanol and treated with RNase AWAY (Molecular BioProducts, San Diego, CA, USA) prior to dissection. After dissection, each tissue was collected directly in a 1.5 mL centrifugal tube, placed on ice, containing 600 µL of RLT buffer of RNeasy Mini kit (Qiagen, Hilden, Germany), and followed by RNA isolation. First, each adult was anesthetized by placing an adult in a 1.5 mL centrifugal tube, and the tube was put on ice for 5 min. The head was isolated by cutting the head from the insect body with microscissors and collected in the RLT buffer. The salivary glands were isolated under the stereomicroscope, the insect was placed on the dissection plate containing 1X phosphate-buffered saline (PBS) and then pinned through the center of the abdomen by an insect-specimen pin. The head was then held with forceps and gently pulled away from the insect body in the horizontal direction. The salivary glands then came out from the foregut along with the head. The salivary glands were then cut off from the head with microscissors and collected in an RLT buffer. Before the isolation of the midgut, first, the legs and wings were cut off, then the head and the salivary glands were removed into the dissection plate containing 1X PBS and placed under the stereomicroscope. The abdomen cavity was then opened by giving a horizontal incision to the abdomen, and the midgut was taken out gently by forceps and collected in RLT buffer. For the remnant body, the head, gut and salivary glands were removed from the insect, and the rest of the body was used for the RNA isolation. The isolated RNA from each tissue was stored at −80 °C until RNA was used as template for cDNA synthesis. In total, four biological replicates were performed in each treatment, with ten pooled midguts, salivary glands, heads and the remnant bodies in each biological replicate. For analysis in different developmental stages, the whole body of 1 to 2 days old nymphs of each developmental stage and adults were used. The insects were collected from the rearing colony and directly processed for total RNA isolation by homogenizing the living individuals in an RLT-buffer + β-mercaptoethanol per the instruction manual of the RNeasy Mini Kit (Qiagen, Hilden, Germany). In total, six biological replicates were performed in each treatment, with six pooled insects in each biological replicate. Total RNA isolation from different tissues and developmental stages and cDNA synthesis were performed as described in the previous section.

The RT-qPCR was performed by a CFX 96™ real-time system (Bio-Rad, Hercules, CA, USA). Two housekeeping genes, *ARP8* and *UBE4A,* were used to normalize the mRNA expression level [38]. *ARP8* and *UBE4A* are responsible for chromatin remodeling and ubiquitin binding during protein recycling, respectively. These two genes have showed the most stable expression (M-value < 0.1 and CV-value < 0.5) across different developmental stages, different tissues and RNAi injections in the brown marmorated stinkbug (*Halyomorpha halys*) [44], which has highly homologous genes with the *N*. *viridula* transcriptome [45]. In addition to *H*. *halys* and *N*. *viridula*, *ARP8* and *UBE4A* have been used as the reference genes in RNAi studies in the Neotropical stinkbug (*Euschistus heros*) [14]. The RT-qPCR specific primers were designed by Geneious Prime^®^ [43] v2020.2.4 software (https://www.geneious.com) (Supplementary Materials Table S2). The RT-qPCR recipe and program were followed as described previously [38]. A Microseal PCR plate (Bio-Rad) was used to set up the RT-qPCR reactions in two technical replicates. The relative normalized mRNA expressions were calculated by the 2^−ΔΔCt^ method [46]. Data analysis was performed as described in the Section 2.7.

### 2.5. “RNAi-of-RNAi”– Oral Feeding Bioassay

To evaluate whether silencing of dsRNase (*NvdsRNase*) can improve oral RNAi efficacy in *N*. *viridula*, dsRNA targeting *NvdsRNase* (ds*Nv*dsRNase) was administered by microinjection to silence *NvdsRNase*. Next, the insects were orally exposed to an artificial diet containing dsRNA targeting an essential gene, *αCop* (ds*αCop*). First, a ds*NvdsRNase* solution (67 nL of 1 µg/µL) was injected at the ventral metathoracic region near the hind coxa in second-instar nymphs (2–3 days old) by microinjection, followed by the feeding of nymphs on their natural diet for 48 h. Microinjection was performed by a nanoinjector (FemtoJet Eppendorf, Hamburg, Germany) with glass needles, self-pulled from 50 µL micropipettes (BRAND GMBH + CO. KG, Wertheim, Germany) by using a PC-100 needle puller (Narishige, Tokyo, Japan). A separate needle was used for each treatment, and each individual was injected with the same needle within a group. After 48 h, when *NvdsRNase* was silenced (determined by *NvdsRNase* silencing dynamics qPCR analysis, Figure S1), nymphs were allowed to feed on sachets containing an artificial diet mixed with ds*αCop* at a final concentration of 300 ng/µL in the artificial diet. In controls, either ds*GFP* or nuclease-free water was used instead of ds*NvdsRNase* (in the microinjection) and ds*αCop* (in the artificial diet). In each control and treatment, 20 nymphs were used, and the whole assay was performed in three independent biological replicates. Preparation of the parafilm-based sachets were performed as described previously [14,38]. Nymphs were allowed to feed on the sachets containing a dsRNA-mixed artificial diet for 5 days and then on the natural diet for the next 9 days. The dsRNA-treated artificial diet was resupplied on the third day, and the natural diet was resupplied every two days. From the 1st day of feeding on the dsRNA treated artificial diet, nymphs were monitored every day for 14 days to observe the phenotypic effects, and the weight of the surviving nymphs was measured as pools of two nymphs with the micro lab balance (Sartorius GMBH, Göttingen, Lower Saxony, Germany), on the 4th, 7th, and 14th day.

To confirm the effect of “RNAi-of-RNAi” at the transcript level, *NvdsRNase* and *αCop* expression levels were quantified in the gut tissues of the 3rd-instar, as 2nd-instar nymphs are too small for the isolation of gut. Third-instar nymphs, injected with either ds*NvdsRNase* or ds*GFP*, fed on the natural diet for 48 h and then continuously fed on the artificial diet containing either ds*αCop* or ds*GFP* for 72 h, as described above in this section. For isolation of total RNA from the gut tissues, the gut was dissected from the insect as follows: first, 3rd-instar nymphs were anesthetized by placing on ice for 2 min and placed in a dissection plate with ventral side up under the microscope. The head was held with forceps and gently pulled out away from the body in a horizontal direction, which led to the removal of the gut and salivary glands which are attached to the head. Total RNA was then isolated from the gut containing the head and the salivary glands. Six pooled guts in each biological replicate with six biological replicates in total were performed for each treatment. RNA was extracted as described in Section 2.3.

### 2.6. “RNAi-of-RNAi”-Ex Vivo Degradation Assay

An ex vivo degradation assay was performed to evaluate the effect of *NvdsRNase*-silencing on the *NvdsRNase* activity in saliva and midgut juice. First, a ds*NvdsRNase* solution (3 µL of 1 µg/µL) was injected in between the third and fourth sterna of the abdomen of each of 20–25 adults (2–3 days old), and insects were allowed to feed on the natural diet for 72 h. Microinjections were executed as described in the previous section, except that here dsRNA was injected in the adults. After 72 h feeding on the natural diet, saliva and midgut juice were collected from the injected adults as described previously [38]. In the control, saliva and midgut juice were collected from ds*GFP*-injected adults. *NvdsRNase* activity was evaluated at 10 min, 30 min, 60 min, and 120 min post-incubation of ds*αCop* with saliva or midgut juice. The procedure of ex vivo degradation assay was as described previously [38].

### 2.7. Statistical Analysis

Survival curves were analyzed by the Kaplan–Meier method with GraphPad Prism v8.4.3 software (San Diego, CA, USA). A log-rank (Mantel–Cox) test and Gehan–Breslow–Wilcoxon test were used to determine the significant difference among the curves (*p*  <  0.05). The Bonferroni method (*p*  <  0.05) was used for multiple comparisons of survival curves. For the RT-qPCR assays, first, normality and equal variance of the dataset were analyzed by the Shapiro–Wilk test and Brown–Forsythe test, respectively. Based on the analysis, statistical differences among the different treatments were determined by Welch’s *t*-test using GraphPad Prism v8.4.3 software (San Diego, CA, USA).

## 3. Results

### 3.1. Identification and Characterization of NvdsRNase

**Figure 1:**
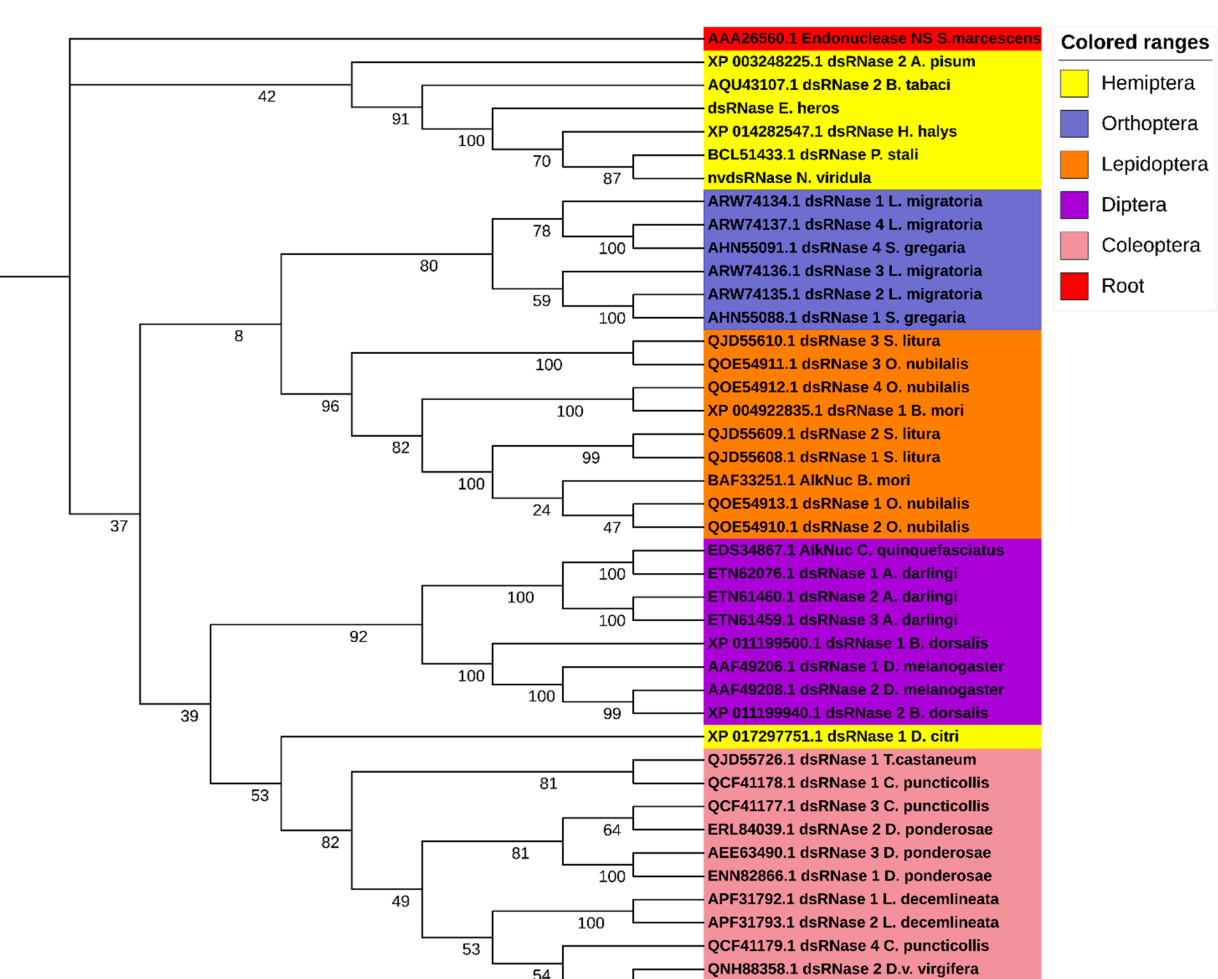
Phylogenetic tree of insect dsRNases, including predicted dsRNase in *N*. *viridula*; *NvdsRNase*, with functionally confirmed homologous *dsRNAses* in other stinkbugs and hemipterans. Amino acid sequences of dsRNases from different insect orders are grouped in different colours. A maximum likelihood algorithm was used to construct the tree with 1000 replicates with a root sequence of Endonuclease_NS conserved domain amino acid sequence from *Serratia marcescens*. Accession numbers of dsRNases are given in the phylogenetic tree.

**Figure 2:**
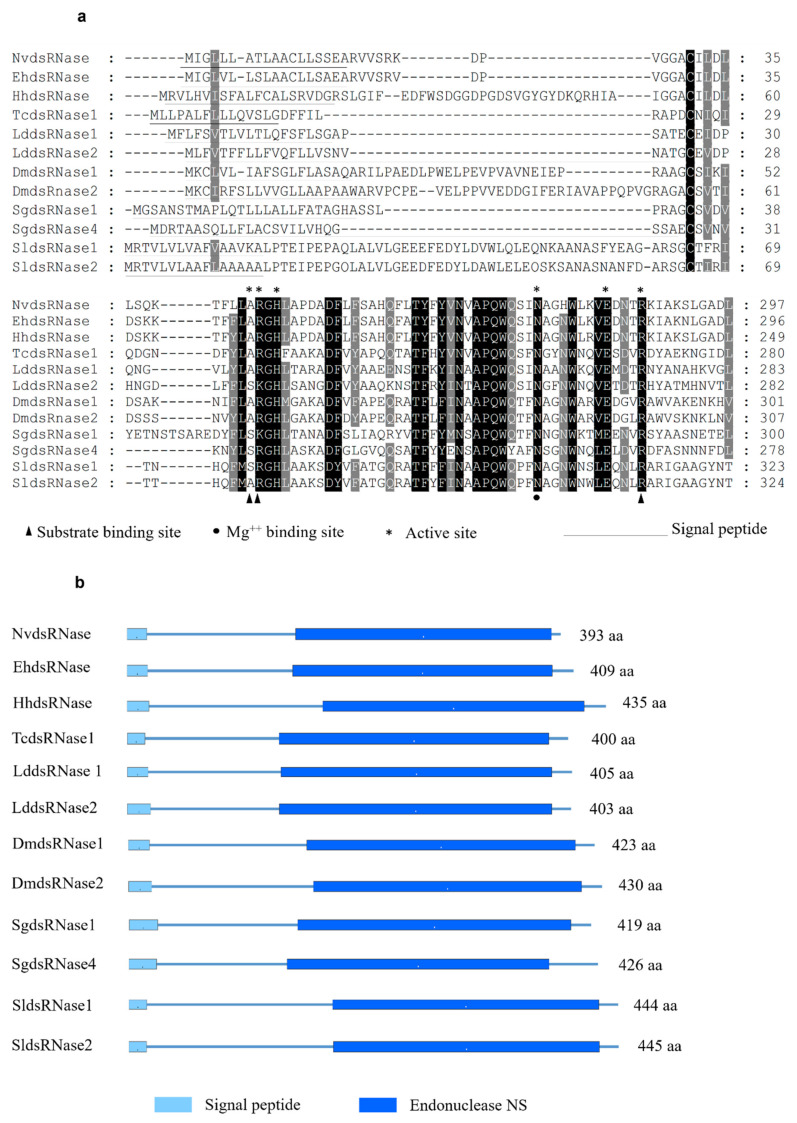
(**a**) Multiple sequence alignment of dsRNases from different insect species: *NvdsRNase* (*Nezara viridula*); *EhdsRNase* (*Euschistus heros* [39]); *HhdsRNase* (*Halyomorpha halys*, XP_014282547.1); *TcdsRNase1* (*Tribolium castaneum*, *QJD55726*.*1)*; *LddsRNase1* (*Leptinotarsa decemlineata APF31792*.*1*); *LddsRNase2* (*L*. *decemlineata*, *APF31793*); *DmdsRNase1* (*Drosophila melanogaster*, *AAF49206*.*1*); *DmdsRNase2* (*D*. *melanogaster*, *AAF49208*.*1*); *SgdsRNase4* (*Schistocerca gregaria*, *AHN55091*.*1*); *SgdsRNase1* (*S*.*gregaria*, *AHN55088*.*1*); *SldsRNase1 (Spodoptera litura*, *QJD55608*.*1*); *SldsRNase2 (Spodoptera litura*, *QJD55609*.*1*). Black and grey highlighted residues are conserved and similar, respectively. Signal peptides are underlined. Active sites are marked by a star, the triangle marks substrate binding sites and the circle marks Mg^++^ binding site. (**b**) Domain arrangement in the aligned amino acid sequences of dsRNase protein in species from different insect orders.

In order to identify the putative dsRNases in *N*. *viridula*, a BLASTn search against the SRA (Sequence Read Archive) database of *N*. *viridula* (SRA, accession number: SRP119668) was performed using nucleotide sequences of different dsRNAs from a range of insect species as query sequences. This search resulted in a partial sequence of one putative *N*. *viridula* dsRNase sequence. To obtain the full ORF, a PCR was performed using a reversed primer designed based on the in-silico analysis of the SRA database of *N*. *viridula* and a forward primer coming from the homologous sequence in the closely related stinkbug *Halyomorpha halys*. The putative dsRNase, *NvdsRNase*, ORF sequence was identified from the sequencing results of the amplified PCR product, and it showed around 80% amino acid sequence identity to the homologous genes in *H*. *hayls* (XP_014282547.1), *E*. *heros* [39], and *Plautia stali* (BCL51433.1). It was found to be clustered in the clade of hemipterans in a phylogenetic tree constructed with a root of dsRNase (Endonuclease_NS) from *Serratia marcescens* (Figure 1). In the deduced amino acid sequence, a signal peptide was identified (amino acid residues 1–18, Figure 2a), indicating that this protein is secreted by the cells and resides extracellularly, which is further confirmed by the subcellular localization prediction. In addition, a conserved DNA/RNA non-specific endonuclease domain (Endonuclease_NS, SM00892) was predicted by SMART (Figure 2b), and a multiple sequence alignment identified the locations of conserved residues responsible for the active site, Mg^++^ binding site and substrate-binding site in the Endonuclease_NS domain (Figure 2a). The amino acid and nucleotide-sequences of *NvdsRNase* ORF are given in Supplementary Materials (nucleotide and amino acid sequence).

### 3.2. Developmental Stage-Specific and Tissue-Specific Expression of NvdsRNase

**Figure 3:**
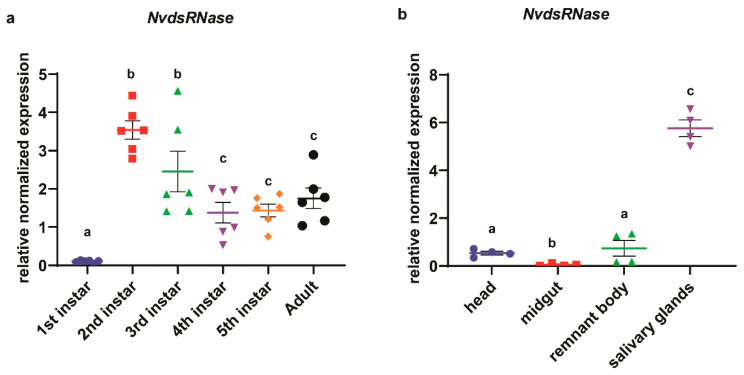
Spatio-temporal expression profile of the *NvdsRNase* gene. (**a**) A scatter plot representation of the mean relative normalized expressions  ±SEM (standard error of mean with six independent biological replicates with 6 pooled insects in each replicate) of the *NvdsRNase* gene in different stages of *N*. *viridula*. Significant differences were calculated by Welch’s *t*-test (*p* > 0.05). (**b**) A scatter plot representation of the mean relative normalized expressions ± SEM (standard error of mean with 4 independent biological replicates with pooled tissues from 10 adults in each replicate) of the *NvdsRNase* gene in different tissues of *N*. *viridula* adults. Significant differences were calculated by Welch’s *t*-test (*p* > 0.05).

In the analysis of the developmental stage-specific expression profile of *NvdsRNase* (Figure 3a), the highest mean expression was observed in the 2nd-instar nymphs, which was significantly different from 4th-, 5th-instar and adults (Figure 3a, Welch’s *t*- test: *p* < 0.05). The lowest mean expression was observed in the newly hatched 1st-instar nymphs which was significantly different from all other developmental stages (Figure 3a, Welch’s *t*-test: *p* < 0.05). In the tissue-specific expression analysis, the highest mean expression was observed in the salivary glands, which was significantly different from the head, midgut and the remnant body (Figure 3b; Welch’s *t*-test: *p* < 0.0001).

### 3.3. “RNAi-of-RNAi”– Oral Feeding Bioassay

**Figure 4:**
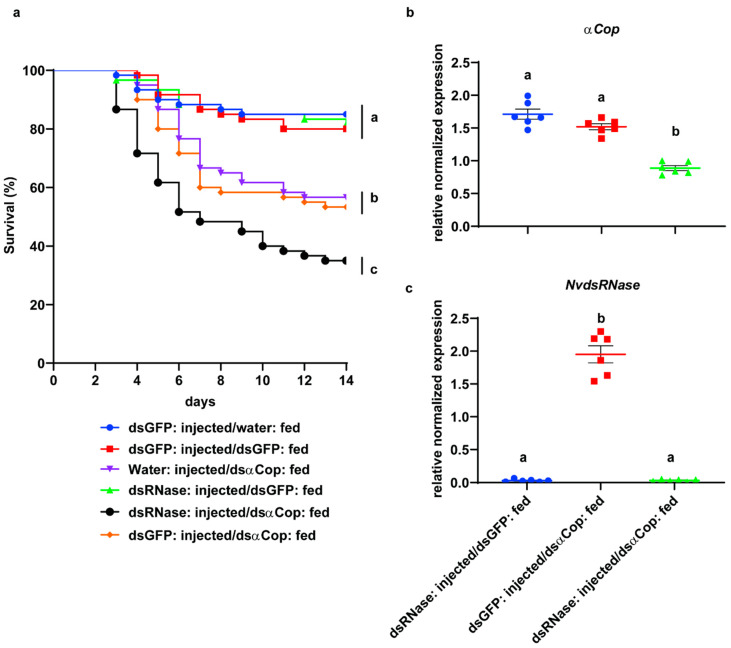
(**a**) Survival curves of 2nd-instar *NvdsRNase*-silenced nymphs from three independent biological replicates after feeding on the ds*αCop* treated artificial diet for 5 days and subsequently for 9 days on the natural diet. Nuclease-free water and green fluorescent protein (GFP) were used as negative controls. Curves that terminate at the different vertical bar are significantly different according to the log-rank (Mantel–Cox) test followed by the Bonferroni test (*p* < 0.05). (**b**,**c**). A scatter plot representation of the mean relative normalized expressions ± SEM (standard error of mean with six independent biological replicates with six pooled guts in each replicate) of the (**b**) *αCop* and (**c**) *NvdsRNase* genes in 2nd-instar ds*NvdsRNase*-injected *N*. *viridula* nymphs at 72 h post continuous feeding on the ds*αCop* mixed artificial diet. Significant differences were calculated by Welch’s *t*-test (*p* > 0.05).

A feeding bioassay was performed to evaluate oral RNAi efficacy in *NvdsRNase*-silenced insects. In this assay, after silencing *NvdsRNase* by injecting the ds*NvdsRNase* solution, the nymphs were fed for 5 days on an artificial diet containing ds*αCop* and on a natural diet for the next 9 days. After 14 days, the ds*NvdsRNase*-injected nymphs feeding on a ds*αCop* mixed diet (ds*NvdsRNase* injected and ds*αCop* fed), showed a mean mortality of 65% which was significantly different from the controls: ds*NvdsRNase* injected and ds*GFP* fed (18.33%), ds*GFP* injected and ds*αCop* fed (46.67%), water injected and ds*αCop* fed (43.33%), ds*GFP* injected and water fed (15.0%), and ds*GFP* injected and ds*GFP* fed (17.5%) (Figure 4a, Log-rank-Mantel-Cox test: *p* < 0.05). In addition to this, the first significant mortality among all the treatments was observed on the 7th day in the ds*NvdsRNase*-injected and ds*αCop*-fed treatment (51.67%), which was significantly different from the water-injected and ds*αCop*-fed treatment (35.0%), but was not significantly different from the ds*GFP*-injected and ds*αCop*-fed treatment (41.5%) (Figure S2, Tukey’s test: *p* < 0.05).

Phenotypic effects were observed every day for 14 days in this assay. A significant reduction in weight was observed on the 7th day in the nymphs that were fed on the ds*αCop* mixed artificial diet (ds*NvdsRNase* injected and ds*αCop* fed, ds*GFP* injected and ds*αCop* fed, and water injected and ds*αCop* fed), compared to the nymphs that were feeding on a non-ds*αCop* treated diet (ds*NvdsRNase* injected and ds*GFP* fed, ds*GFP* injected and water fed, and ds*GFP* injected and ds*GFP* fed) (Figure S3, Tukey’s test: *p* < 0.05). However, there was no significant difference between the nymphs in ds*NvdsRNase* injected and ds*αCop* fed, ds*GFP* injected and ds*αCop* fed, and water injected and ds*αCop* fed (Figure S3).

An RT-qPCR assay was performed to evaluate the effect of “RNAi-of-RNAi” at the transcript level. The transcript levels of *NvdsRNase* and the essential gene *αCop* were quantified at 72 h post continuously feeding on an artificial diet mixed with ds*αCop*. The expression of *NvdsRNase* was efficiently downregulated, as a 98% reduction in the transcripts of *NvdsRNase* was observed in the ds*NvdsRNase*-injected and ds*αCop*-fed treatment compared with the ds*GFP*-injected control (ds*GFP* injected and ds*αCop* fed) (Figure 4c, Welch’s *t*-test: *p* < 0.05). In the ds*NvdsRNase*-injected and ds*αCop*-fed treatment, a 48% reduction in the transcripts of *αCop* was observed, significantly different from ds*GFP* injected and ds*αCop* fed, and ds*NvdsRNase* injected and ds*GFP* fed (Figure 4b, Welch’s *t*-test: *p* < 0.05).

### 3.4. “RNAi-of-RNAi”-Ex Vivo Degradation Assay

**Figure 5:**
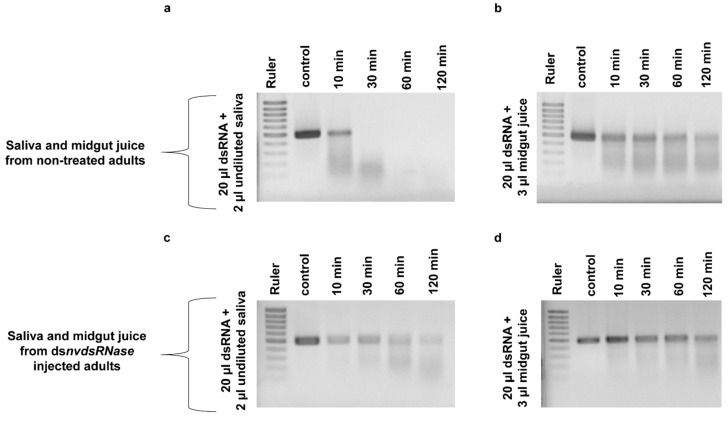
ds*αCop* incubation in undiluted saliva and midgut juice collected from ds*NvdsRNase*-injected (**c**,**d**) and ds*GFP*-injected (**a**,**b**) adults. A prolonged stability of ds*αCop* was observed in the saliva and midgut juice collected from ds*NvdsRNase*-injected adults compared to the ds*αCop* in the saliva and midgut juice collected from ds*GFP*-injected adults.

The effect of *NvdsRNase* silencing on dsRNase-activity in saliva and midgut juice was evaluated in an ex vivo degradation assay. *NvdsRNase* was silenced in adults by injection, followed by saliva and midgut juice collection 72 h later. After incubation of ds*αCop* in saliva collected from ds*NvdsRNase*-injected adults, a partial amount of ds*αCop* was found intact up to 120 min, even though a fraction had already degraded after 10 min (Figure 5c). In contrast, ds*αCop* incubated in saliva collected from ds*GFP*-injected adults degraded more quickly, and a complete degradation was already observed after 30 min (Figure 5a). In the degradation assay with midgut juice, more ds*αCop* remained intact for longer in midgut juice collected from ds*NvdsRNase*-injected adults than the midgut juice collected from ds*GFP*-injected adults (Figure 5b,d).

## 4. Discussion

In previous studies on *N*. *viridula*, microinjection-delivered dsRNA resulted in a sensitive RNAi response in contrast to orally delivered dsRNA, where the oral delivery route showed a significantly lower RNAi response [38]. This phenomenon is not uncommon and has been observed in various species from different insect orders [14,20,21]. Multiple factors have been reported that impair the oral RNAi response, including the efficiency of cellular uptake of dsRNA, the tissue of the target gene expression, duration of dsRNA feeding, and dsRNAase activity in the digestion canal or haemolymph [18]. In the present study, we identified and characterized a dsRNAase negatively affecting oral RNAi efficacy in *N*. *viridula*. The core RNAi machinery’s gene expression and RNAi functionality in *N*. *viridula* has been reported in different studies [47,48,49]. In our previous study, RNAi response to the orally delivered dsRNA was demonstrated, where the *αCop* gene led to the highest mortality in *N*. *viridula* nymphs [38], suggesting that *αCop* is a suitable gene for this assay. However, we also observed rapid degradation of dsRNA by digestive fluids in this previous study.

In this study, only one dsRNase was identified in the transcriptome of *N*. *viridula*. Similarly, in other stinkbugs, *H*. *hayls* and *E*. *heros*, only one dsRNase has been identified and is included in the phylogenetic tree [39] (Figure 1). Homoptera on the other hand, another group of hemipterans, appear to express multiple dsRNA-degrading nucleases. For example, in *Bemisia tabaci* [30], two dsRNases were found to be expressed, and in the genome of *Acyrthosiphon pisum*, also two dsRNase-encoding genes have been predicted (dsRNase1: XP_003242652.1, dsRNase2: XP_003248225.1). Additionally, in these Homoptera, there are reports of low sensitivity to oral RNAi, with variability between species [22,30,50]. In other insect orders such as Coleoptera, Orthoptera and Lepidoptera, three to four dsRNases have been found to be expressed [23,24,27]. The characterization of the *NvdsRNase* amino acid sequence delineated the presence of the Endonuclease_NS (SM00892) domain and a signal peptide, specific to the extracellular secretory pathway, indicating the involvement of this protein in an extracellular dsRNA degradation. Multiple sequence alignments revealed that the critical amino acid residues in *NvdsRNase* are similar to those in other stinkbugs (Figure 2a). It also identified the substrate-specificity of this protein, as the active site shares the common residues with *LmdsRNase1* (*Locusta migratoria*), which is known to have substrate-specificity to dsRNA and dsDNA [51] (Figure 2a).

In the analysis of *NvdsRNase* expression in different developmental stages, a variable expression was observed across the different stages (Figure 3a). Interestingly, the mean expression of *NvdsRNase* in newly hatched 1st-instars was the lowest and showed a huge difference compared to the expression in the 2nd-instar. An explanation for this observation could be the feeding habits of *N*. *viridula*, as hatched nymphs do not start feeding on plants until the 2nd-instar [52]. In 4th-, 5th-instar and adults, a relatively lower expression was observed but significantly different from the 2nd- and 3rd-instar, indicating *NvdsRNase* perhaps overexpresses in 2nd-instar when nymphs begin feeding on the plants and gradually reduces in the later developmental stages as more proteins are present (Figure 3a). In the tissue-specific expressions of *NvdsRNase* (Figure 3b), the mean expression was the highest in salivary glands, and a huge difference in the expression level in the salivary glands tissue and other tissues (the head, midgut and remnant body) suggested that the salivary glands are the key source of dsRNase-protein synthesis that limits oral RNAi efficacy in *N*. *viridula*.

To evaluate the effect of *NvdsRNase* knockdown on oral RNAi efficacy in *N*. *viridula*, *NvdsRNase* was silenced by injecting 67 ng of ds*NvdsRNase* solution into 2nd-instar nymphs, which was followed by the feeding of nymphs on an artificial diet containing ds*αCop*. A significant difference in the mortality was observed between the ds*NvdsRNase*-injected and ds*αCop*-fed treatment and the controls (ds*GFP* injected and ds*αCop* fed, and water injected and ds*αCop* fed). This result indicates that the suppression of dsRNase significantly improves oral RNAi efficacy in *N*. *viridula*. These results are similar to the previous study in another hemipteran, *B*. *tabaci*, where elevated mortality was observed in the adults after exposure to a diet containing dsRNAs specific to a lethal target gene and to the dsRNases genes [30]. Similar findings have been observed in other insect orders: Coleoptera [23,25], Diptera [26] and Orthoptera [29]. This confirms that dsRNases play a role in RNAi efficiency in a wider range of insects. In addition to this, there was no significant mortality in the control where ds*NvdsRNase* was injected compared to the ds*GFP*-injected control, indicating that it is not an essential gene. Indeed, previous studies have identified the role of dsRNase protein in the antiviral defense in species from various insect orders [7]. 

In our previous study, an ex vivo dsRNA degradation assay in undiluted saliva has shown a quick degradation of ds*αCop* [38], which supported the hypothesis of dsRNase activity as one of the most critical factors in limiting RNAi efficacy [12,14,21,25]. In a further investigation to evaluate the effect of “RNAi-of-RNAi” on *NvdsRNase* activity, an ex vivo dsRNA degradation assay was performed. Ds*αCop* in undiluted saliva, collected from ds*NvdsRNase*-injected insects, showed prolonged stability compared to the control (Figure 5), implying that *NvdsRNase* silencing significantly improves dsRNA stability in saliva. However, ds*αCop* in the ds*NvdsRNase*-injected insect saliva seems to be degraded over 10 min to 120 min (Figure 5), which can be attributed to the mRNA expression level and the protein half-life of *NvdsRNase* [53]. A higher or longer knockdown of *NvdsRNase* is possibly required prior to saliva collection, which could further improve ds*αCop* stability.

A successful dsRNA delivery via the oral route is a prerequisite to develop RNAi technology as a biopesticide strategy. Among all the insect orders, dsRNase activity in the oral route is one of the decisive hindrances in applying this technology at the field level. However, dsRNase activity varies across the different orders depending on the types and localization of the dsRNase protein. For example, the lepidopteran *Spodoptera litura* is one of the most RNAi-recalcitrant insects in which RNAi fails to work by feeding and by injection; six types of dsRNases have been identified across different tissues including midgut and haemolymph [24]. Such insects are the most challenging pests to control by RNAi technology [13]. In contrast, pests exhibiting systemic RNAi (coleopterans) have already been demonstrated to be efficiently controlled by transgenic plants expressing an insect-specific dsRNA [54]. Transgenic crops have their own social and technical constraints, narrowing the acceptability/applicability of genetically modified crops in different regions across the globe [11]. Therefore, in such regions, non-transformative strategies have to be explored: these include spray-induced gene silencing (SIGS), root drenching, foliar spray, trunk injections, etc. [55]. Apart from the dsRNase activity, other factors influencing the RNAi efficacy in non-transformative strategies are the half-life period of dsRNA in the environment and various barriers in dsRNA uptake by the plants [8]. Hence, the use of formulations and delivery carriers to prolong dsRNA persistence and improve efficacy are required. In recent reports, several companies have claimed the development of co-formulants to overcome the hurdles mentioned above [56]. To protect dsRNA in the gut, various nanoparticles and peptide-based formulations have shown the successful enhancement of RNAi efficacy [57,58]. The feeding mechanism of *N*. *viridula* includes feeding on xylem and cell sap [59], allowing it to be targeted by SIGSs. Therefore, simultaneous use of dsRNase-specific and the target gene-specific-dsRNAs could be a potential strategy that could further enhance oral RNAi efficacy in the field. In a previous study, the combined use of dsRNAs has shown an improved oral RNAi efficacy in *B*. *tabaci* [30]. However, in order to apply this technology at the field level, it will be beneficial in the future to screen the adults of *N*. *viridula* to assess the efficacy of combined use of dsRNAses and essential target genes. These kinds of improvements could contribute to the development of efficacious RNAi-based biopesticides for sucking pests.

## 5. Conclusions

In conclusion, the present study identified and characterized a dsRNase: *NvdsRNase* in *N*. *viridula*. We provided critical information and identified the role of *NvdsRNase* in limiting oral RNAi efficacy, and that the silencing of *NvdsRNase* improves the stability of the dsRNA in the saliva and midgut juice. Significantly higher mortality in the *NvdsRNase*-silenced nymphs proves that suppression of such genes can enhance RNAi efficacy in *N*. *viridula*. For practical applications at the field level, the concurrent use of dsRNA targeting *dsRNase* and dsRNA targeting a lethal essential gene, combined with suitable delivery carriers, could be a way forward for a successful pest control strategies.

## Data Availability

Data is contained within the article or supplementary material.

## Supplementary Materials

The following are available online at https://www.mdpi.com/2075-4450/12/2/115/s1. Figure S1: Each bar shows the mean relative normalized expressions ± SEM (standard error of mean with three independent biological replicates) of the nvdsRNase gene in 2nd-instars of N. viridula at 48 h, 72 h, 96 h and 120 h post injections of dsnvdsRNase, dsGFP was used as a negative control. *P*-values were calculated by unpaired multiple *t*-test (*p* < 0.05), Figure S2: Each bar shows the mean mortality ± SEM (standard error of mean with two independent biological replicates) of 2nd-instars nvdsRNase-silenced nymphs on (a) 7th and (b) 14th day after feeding on dsαCop treated artificial diet for 5 days and subsequently for 9 days on the natural diet. Significant differences among the treatments were calculated by one way ANOVA and followed by Tukey’s test (*p* < 0.05), Figure S3: Each bar shows the mean body weight ± SEM (standard error of mean with two independent biological replicates) of 2nd-instar nvdsRNase-silenced nymphs on (a) 4th, (b) 7th and (c) 14th day after feeding on dsαCop treated artificial diet for 5 days and subsequently for 9 days on the natural diet. Significant differences among the treatment were calculated by one way ANOVA followed by Tukey’s test (*p* < 0.05), Table S1: Nucleotide sequence of NvdsRNase ORF, Table S2: Amino acid sequence of NvdsRNase ORF.
