# Supplementary material for: Silencing of Double-Stranded Ribonuclease Improves Oral RNAi Efficacy in Southern Green Stinkbug Nezara viridula
Source: Insects. 2021 Jan 28;12(2):115. doi: 10.3390/insects12020115 (PMC7912330; doi:10.3390/insects12020115)
Supplement: Supplementary file 1 [file insects-12-00115-s001.pdf]

Article

Silencing of Double-Stranded Ribonuclease Improves Oral RNAi Efficacy in Southern Green Stinkbug *Nezara viridula*

Rohit Sharma, Clauvis Nji Tizi Taning, Guy Smagghe and Olivier Christiaens *

Department of Plants and Crops, Faculty of Bioscience Engineering, Ghent University, Coupure Links 653, B-9000 Ghent, Belgium; rohit.sharma@ugent.be (R.S.); tiziclauvis.taningnji@ugent.be (C.N.T.T.); guy.smagghe@ugent.be (G.S.)

* Correspondence: olchrist.Christiaens@UGent.be, Tel.: +32-9-264-6144

Supplementary data:

Table S1. Nucleotide sequence of *NvdsRNase* ORF

NvdsRNase Nezara viridula
ATGATTGGACTTCTTCTTGCGACGCTTGCGGCGTGCCTTCTCTCTTCGGAGGCGAGGGTCG TTTCTAGGAAAGATCCTGTCGGGGGCGCTTGTATTCTGGACTTGAATACTGACTTGCCGAAGA AAAATGAACCTCTTTTTCTTCAACGATCTCCTTCCGGATCCTTAGACCTGGTCTTGCCGGAAAT GGAAGGAAAACGAGGAGTCATTGCTCTCAGGGAAGGCGAGCAAATCTTGATTTCTTGTCCTG GCAAGAAGAACCACCTCGCTATCACTAACAGTGAAGCTTCCGGAGCTTCCTGCAAGGCTGGA AAGACCTTATCCATCGATGGATCCGACTATTCTTCTCAAGACCTCGACTGTAGCTCCAGGGCT GGCTCCACCACCAGACCTACCCAGAAGAAGTGTGCTGGAGGAAAGGGAATAATCGTTGAAC TGGGATTCGATGTCGAAGATTCGTGGATTCCGATGATCGAGACCTGCCACGACGTTGAGAAC AGCAACTCCTTCTACTCCGTCCACACCATCCACGGCGCCATCATGGGAGGCAAGGTCTACAG GACTACCGCGAGACCACTCTTCGCCAGGGGAGACAGCATCTTCTTCAAGGGATTCAATCCAG AGCACGCCTATGCCCAGAAAAACCAAAAGGACGTCCTCGCCCGTGAACTTGGAGCAGCCAA CGCCAACAAATACCTTCTCTCTCAGAAAACTTTCCTCTTGGCCAGAGGACATCTCGCACCTGA CGCAGATTTCCTTTTCAGCGCTCATCAGTTTTTGACCTACTTCTACGTCAACGTAGCTCCACAA TGGCAATCTATCAACGCCGGTCATTGGCTCAAGGTAGAAGACAACACAAGGAAAATCGCCA AGAGTCTCGGTGCTGACCTTCAAGTAGTCACCGGAACCGAAGGAGTCCTCACCTTGCCAGCA GCCAGAGGAGTGAAGGAGATCAAGCTCCAGGGCTCCAGACTTCCTGTACCAAATCACTTCTG GAAGGTCCTCAGGAACACCCAAGACGATTCCTGCATTGCCTTCGTCTCCACAACAACCCCTTT CTTACAAGCCCACCCAGGACCTTCTGCAAGGACATCTGCGCTGAAAGTAAATGGCCTAAGCT CCAAGACGACTTATCCAAAGCTACGTAAACGGCTGCCGATACAAAGACCTCAATCTTTCTAG

Table S2. Amino acid sequence of *NvdsRNase* ORF

NvdsRNase Nezara viridula
MIGLLLATLAACLLSSEARVVSRKDPVGGACILDLNTDLPKKNEPLFLQRSPSGSLDLVLPEME GKRGVIALREGEQILISCPGKKNHLAITNSEASGASCKAGKTLSIDGSDYSSQDLDCSSRAGSTTRPT QKKCAGGKGIIVELGFDVEDSWIPMIETCHDVENSNSFYSVHTIHGAIMGGKVYRTTARPLFARGDS IFFKGFNPEHAYAQKNQKDVLARELGAANANKYLLSQKTFLLARGHLAPDADFLFSAHQFLTYFY VNVAPQWQSINAGHWLKVEDNTRKIAKSLGADLQVVTGTEGVLTLPAARGVKEIKLQGSRLPVPN HFWKVLRNTQDDSCIAFVSTTTPFLQAHPGPSARTSALKVNGLSSKTTYPKLRKRLPIQRPQSF*

Supplementary figures:

Figure S1

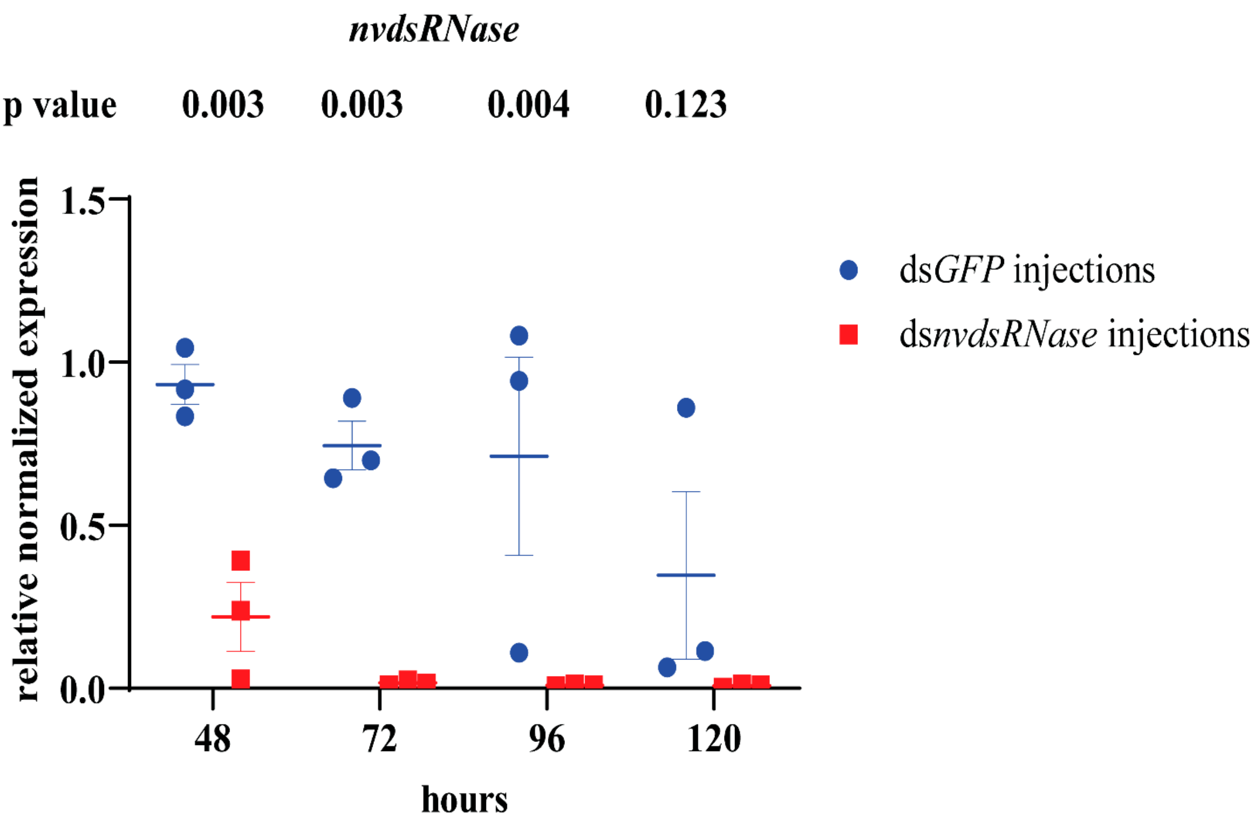

Figure S1. Each bar shows the mean relative normalized expressions ± SEM (standard error of mean with three independent biological replicates) of the *nvdsRNase* gene in 2nd-instars of *N. viridula* at 48 h, 72 h, 96 h and 120 h post injections of ds*nvdsRNase*, ds*GFP* was used as a negative control. P-values were calculated by unpaired multiple *t*-test ($P < 0.05$).

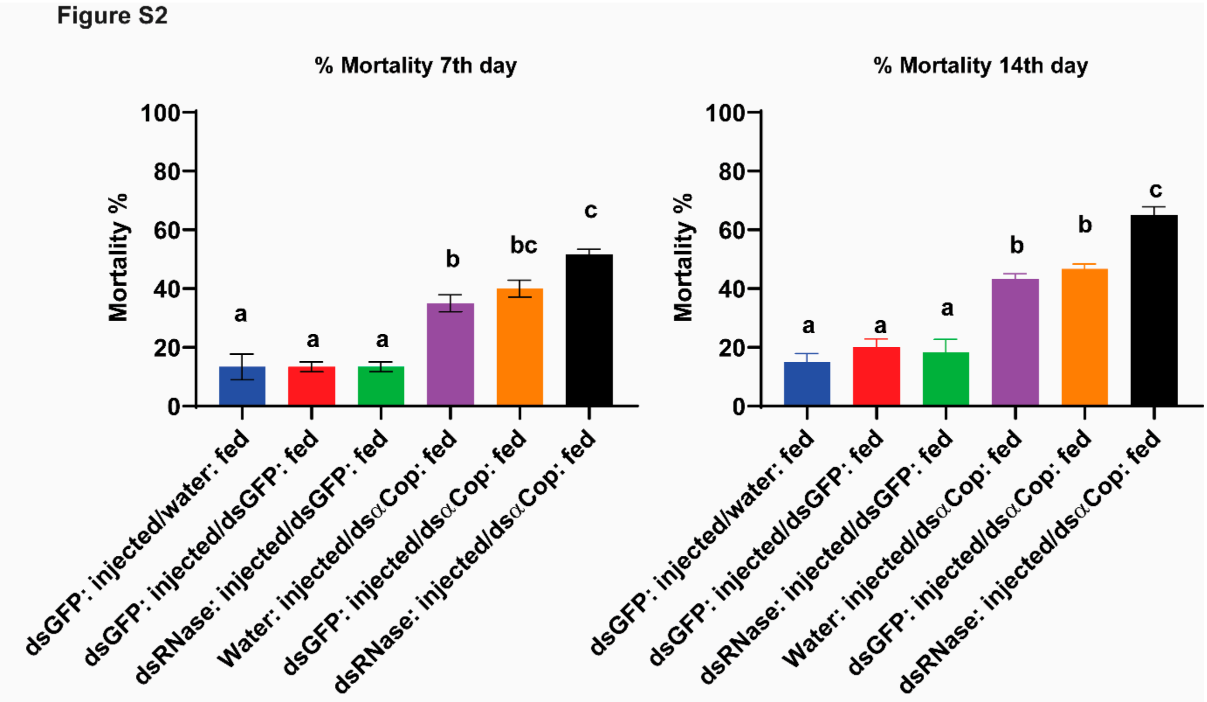

Figure S2. Each bar shows the mean mortality ± SEM (standard error of mean with two independent biological replicates) of 2nd-instars *nvdsRNase*-silenced nymphs on (**a**) 7th and (**b**) 14th day after feeding on ds*αCop* treated artificial diet for 5 days and subsequently for 9 days on the natural diet. Significant differences among the treatments were calculated by one way ANOVA and followed by Tukey's test ($P < 0.05$).

Figure S3

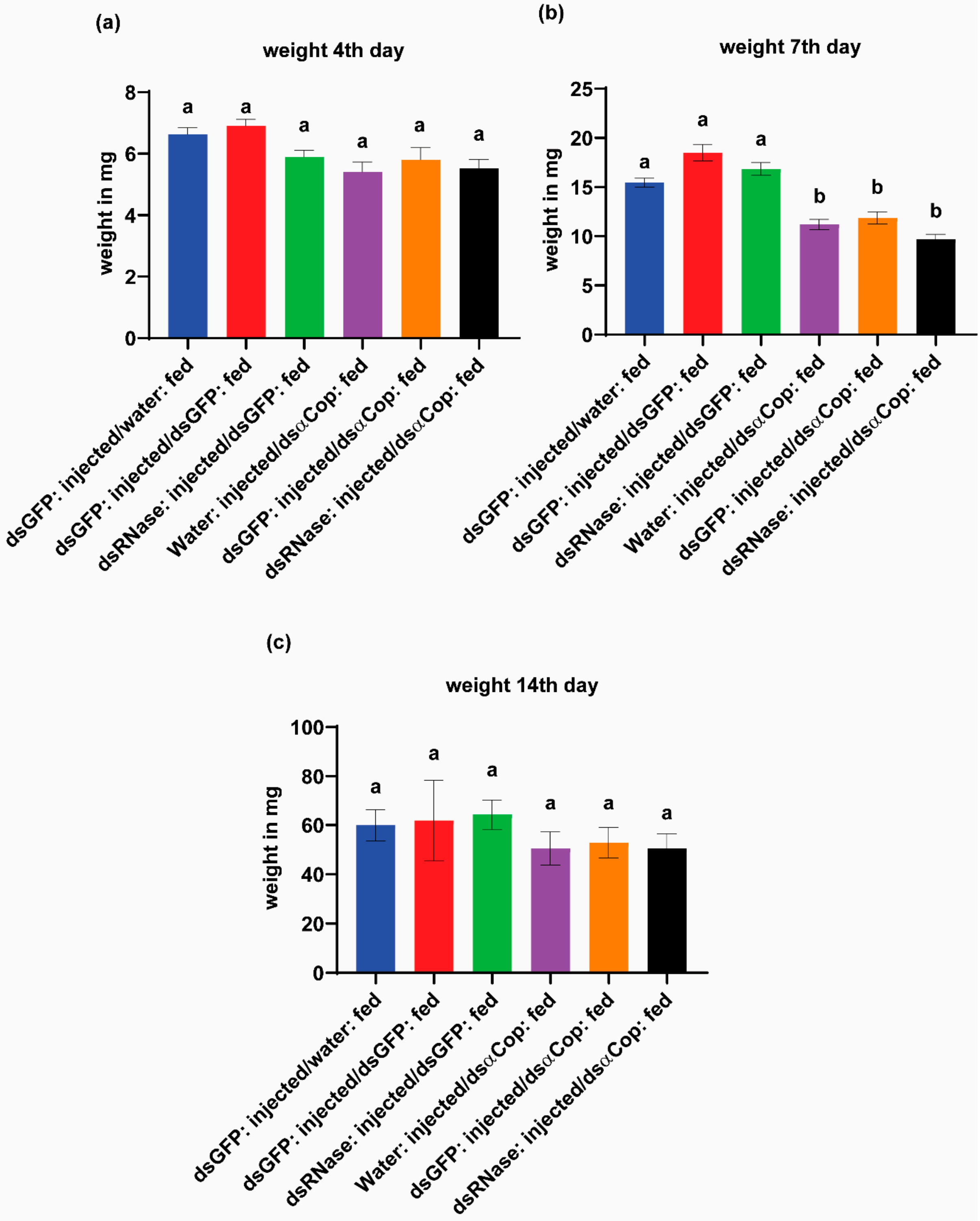

Figure S3: Each bar shows the mean body weight ± SEM (standard error of mean with two independent biological replicates) of 2nd-instar *nvdsRNase*-silenced nymphs on (**a**) 4th, (**b**) 7th and (**c**) 14th day after feeding on ds*αCop* treated artificial diet for 5 days and subsequently for 9 days on the natural diet. Significant differences among the treatment were calculated by one way ANOVA followed by Tukey's test ($P < 0.05$).
